# Synthesis, Properties, and Biological Applications of Metallic Alloy Nanoparticles

**DOI:** 10.3390/ijms21145174

**Published:** 2020-07-21

**Authors:** Kim-Hung Huynh, Xuan-Hung Pham, Jaehi Kim, Sang Hun Lee, Hyejin Chang, Won-Yeop Rho, Bong-Hyun Jun

**Affiliations:** 1 Department of Bioscience and Biotechnology, Konkuk University, Seoul 143-701, Korea; huynhkimhung82@gmail.com (K.-H.H.); phamricky@gmail.com (X.-H.P.); susia45@gmail.com (J.K.); 2 Department of Bioengineering, University of California, Berkeley, CA 94720-1762, USA; shlee.ucb@gmail.com; 3 Division of Science Education, Kangwon National University, Chuncheon 24341, Korea; hjchang@kangwon.ac.kr; 4 School of International Engineering and Science, Jeonbuk National University, Jeonju 54896, Korea; rho7272@jbnu.ac.kr

**Keywords:** alloy nanoparticles, bimetallic nanoparticles, trimetallic nanoparticles, biological application, synergistic effect, photocatalytic property, super-magnetism

## Abstract

Metallic alloy nanoparticles are synthesized by combining two or more different metals. Bimetallic or trimetallic nanoparticles are considered more effective than monometallic nanoparticles because of their synergistic characteristics. In this review, we outline the structure, synthesis method, properties, and biological applications of metallic alloy nanoparticles based on their plasmonic, catalytic, and magnetic characteristics.

## 1. Introduction

In principle, nanomaterials indicate materials from subnanometer to several hundred nanometers in size that are applied in material science and nanotechnology due to properties different from those of conventional materials. Metallic nanoparticles are small particles made of metal and can be synthesized by physical, chemical, or biological-based methods. Their properties depend on their composition, size, and shape that determine their plasmonic, catalytic, and magnetic characteristics. Monometallic nanoparticles obtain the property of their constituent metal whereas bimetallic and trimetallic alloy nanoparticles synthesized from two or three metals show more stable structures and enhanced properties. Additionally, alloy nanoparticles demonstrate synergistic effects due to hybrid characteristics such as photocatalytic properties and super-magnetism. Therefore, alloy nanoparticles are being progressively studied for potentially diverse applications. In this article, we summarized and reviewed the results of scientific research over the last 10 years which highlighted the role of alloy nanoparticles in biological applications such as bio-imaging, sensors, catalyst, drug delivery, and therapies as well as their types, synthesis, and properties.

## 2. Classification of Alloy Nanoparticles

According to atomic ordering, bimetallic nanoparticles can be classified into four types [1,2]:

### 2.1. Mixed Alloyed Nanoparticles

**Figure 1:**
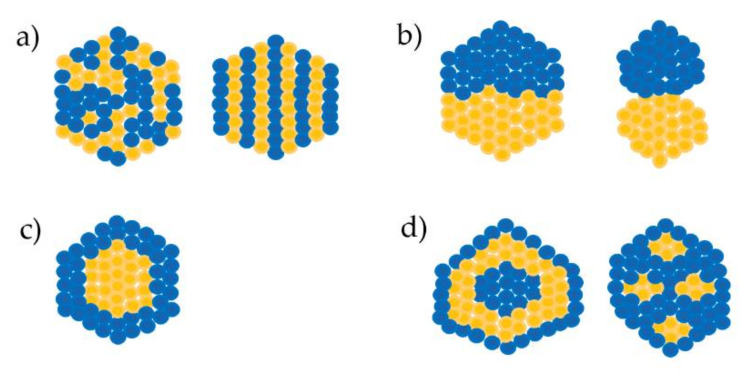
Types of bimetallic alloyed nanoparticles. (**a**) Mixed alloyed nanoparticles; (**b**) Sub-cluster segregated alloyed nanoparticles; (**c**) core-shell alloyed nanoparticles; (**d**) multiple core–shell alloyed nanoparticles [1,2].

They may have a random or ordered arrangement (Figure 1a). Randomly mixed nanoalloys are often termed alloyed nanoparticles, whereas ordered mixed nanoalloys are termed intermixed or intermetallic nanoparticles [3].

### 2.2. Sub-Cluster Segregated Alloyed Nanoparticles 

These nanoparticles comprise two small clusters (sub-clusters) in their structure (Figure 1b). There are two kinds of sub-clusters. One shares the middle interface whereas the other only shares a bone or short interface between two small clusters [4].

### 2.3. Core-Shell Alloyed Nanoparticles 

These nanoparticles typically consist of one metal that forms a shell surrounding a core made of another metal or the recent core–shell type which is composed of an intermixed core surrounded by a pure shell [5]. This type is created more commonly and has diverse applications [6] (Figure 1c).

### 2.4. Multiple Core-Shell Alloyed Nanoparticles 

These have two further kinds of multiple arrangements, multiple shell–core nanoparticles created with two or more shells covering a single core, and multiple core–shell nanoparticles with one simple shell surrounding several cores; the shell and core are always composed of two different metals [7,8,9,10,11,12] (Figure 1d).

The degree of mixing and atomic ordering in bimetallic nanoparticles can be controlled by the relative strength of the bond between two chemicals, the surface energy, atomic size, electric or magnetic effects, etc. [1].

Trimetallic nanoparticle types are similar to those of bimetallic nanoparticles. These are mixed alloyed nanoparticles with a mixture of three metals and core–shell alloyed nanoparticles with a mixture of two kinds of chemicals in the core with a single chemical in the shell or simply one chemical in the core with a mixture of two chemicals in the shell. Another type of trimetallic nanoparticles is the separate core–shell type in which each of the three chemicals sequentially form the core–shell–shell [13,14,15,16,17,18,19,20].

## 3. Methods of Synthesizing Alloy Nanoparticles

There are two basic ways to synthesize nanoparticles: the top-down approach and bottom-up approach. The top-down approach involves the production of nanoparticles from macro-sized materials, whereas the bottom-up approach involves the creation of nanoparticles from atoms. Between both methods, the bottom-up method is more popular and developed, and generally relies on synthesis pathways of two main categories: simultaneous method and successive method. The simultaneous method demands precursor materials of the metals of interest (which can be bimetallic or trimetallic alloy clusters) in the same reaction. The successive method involves the growth of particles by reducing metal ions over the surface of another metal core [2,21].

### 3.1. Physical Methods-Based Nanoparticle Synthesis

#### 3.1.1. Sputtering

Sputtering is a procedure in which nanoparticles are created by bombarding the target metal with high energy [1]. Atom beam sputtering involves three basic steps: migration of atoms from the surface of materials, nucleation and growth of nanoparticles, and absorption onto another material in an electric field [22,23,24]. Magnetron sputtering involves sputtering in a magnetic field, in which one or more materials are deposited on the surface of another material such as metal or ceramics through a high-rate vacuum coating technique [25,26,27,28,29]. This method can provide high purity but it is difficult to control the morphology of the nanoparticles formed; moreover, its energy requirement is too high, which can pose a danger [30]. There are many studies on making thin films with alloy nanoparticles on the surface using the sputtering method, such as Au–Ag alloy nanoparticles in SiO_2_ or TiO_2_ thin films [25,27,28].

#### 3.1.2. Thermal Decomposition

Thermal decomposition-based methods synthesize nanoparticles based on temperature. Synthesis of transition metal nanoparticles such as Fe, Ni, and Co requires high temperatures because these metal nanoparticles are not stable at room temperature. This method is also applied for metals that have low reduction potential or difficult reduction characteristics. The process starts with forming particles of the metal precursor having lower decomposition temperature, followed by the next metal precursor that decomposes when the temperature is increased [2,31,32]. This method is used for fabricating good quality crystals or commercial value crystals. The main disadvantage of this technique is the need for high temperatures, which can be dangerous, and difficulty in isolating unstable nanoparticles from the reaction at high temperature [30,33]. Fe-, Ni-, and Co-based alloy nanoparticles such as Pt–Co, Au–Fe, Au–Ni, Au–Co, Fe–Co, Fe–Ni, Co–Pt, Ni–Mo, Pt–Ni–Fe, Sn–Zn–Cu, and Au–Cu–Pt are produced by thermal decomposition [14,20,34,35,36,37,38,39,40,41,42,43,44].

#### 3.1.3. Radiolytic Method

Fabrication of metal nanoparticles by irradiation is called radiolytic synthesis. In this case, the gamma (γ) ray or electron beam is used to reduce metal ions in soluble precursors thus forming metal nanoparticles. This method can produce alloy nanoparticles that are not stable when created by thermal decomposition. The type of alloy nanoparticle created depends on the dose of irradiation. A low dose can lead to the creation of core–shell alloy nanoparticles whereas a higher dose controls the making of mixed alloy nanoparticles [45,46,47,48]. The difficulty of the radiolytic method is in directing the nanoparticles’ shape. However, irradiation-based techniques are low-cost, environment-friendly, and show promise for large applications [2,49,50,51]. There are many examples of the alloy nanoparticles fabricated based on radiolytic synthesis, such as Rh–Pd, Rh–Pt, Au–Ag, Au–Pt, Au–Pt–Ag, Pt–Ru–Sn, Pd–Ru–Ni, and Zr–Ni–Cu alloy nanoparticles [46,52,53,54,55,56,57,58,59,60,61].

#### 3.1.4. Sonochemical Synthesis

**Figure 2:**
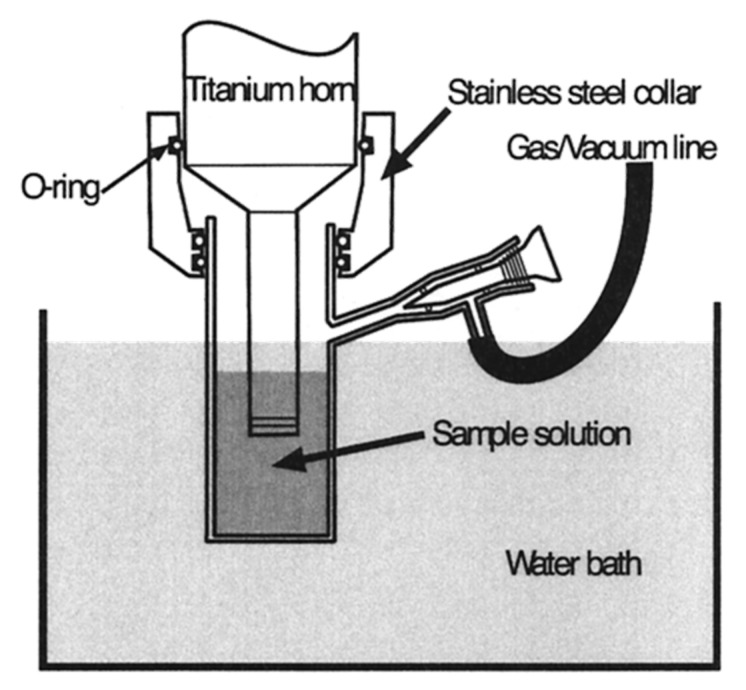
Schematics of the sonication reactor [62].

The sonochemical synthesis method is based on ultrasound. In solution, ultrasound can cause high temperature or high pressure. Due to the increased temperature, small metal nanoparticles are created at a rapid reaction rate. Ultrasound induces the collapse or formation of tiny bubbles in a solution that allows the creation of hollow nanoparticles. Further, the production of oxidizing and reducing radicals is crucial for the synthesis of metal nanoparticles [62,63,64,65]. Figure 2 showed brief schematics of the sonication reactor. Sonochemical synthesis has been in use for more than twenty years to synthesize bimetallic alloy nanoparticles such as Au–Pd nanoparticles [66] and has been recently combined with other techniques for the creation of alloy nanoparticles [67,68]. Various alloy nanoparticles such as Au–Pd, Co–Cu, Fe–Pt, Hg–Pd, Au–Ru, Pt–Cu, Fe–Ag–Pt, Pd–Co–Pt have been manufactured by using sonochemical synthesis method [13,66,67,69,70,71,72,73,74,75].

### 3.2. Chemical-Method Based Nanoparticle Synthesis

#### 3.2.1. Chemical Reduction

The chemical reduction method is used for producing bi-/tri-metallic alloy nanoparticles through the reduction of appropriate precursors to the zero-valent state. This method involves two phases, reduction and growth. The reduction process occurs sequentially: at first, the metal precursors owning to the highest redox potential precipitate to form the core, followed by the second and possibly third precursor being deposited as a shell [1,31,33,76,77]. Organic solvents are used to prevent agglomeration and maintain the stability of nanoparticles in the solution phase [78,79,80,81]. The advantages of the co-reduction technique include simplicity of steps and versatile application but still has the disadvantage of presence of impurities; for instance, the Au-Pd-Pt trimetallic alloy nanoparticle created by simultaneous reduction of multiple metal precursors had an Au core with a mixed Pd and Pt shell that was not separated into two separate layers as the Pd shell and Pt shell [17]. Due to its simplicity, bimetallic (Pt–Ag, Pt–Co, Pt–Au, Pd–Ag, Pd–Pt, Au–Ag, Au–Pt, Ag–Au, Ag–Co) and trimetallic (Pt–Pd–Co, Co–Ni–Cu, Ni–Au–Pd, Pd–Pt–Ni, Au–Pd–Pt) alloy nanoparticles have been produced by this method and will be further improved in future [78,81,82,83,84,85,86,87,88,89,90,91,92].

Galvanic displacement reactions are possibly considered as a chemical reduction subsection that is used to produced bimetallic hollow and porous nanoparticles [93]. This method is the replacement reaction which is based on electrical potential difference between two metals that are templates and a salt precursor in a suspension [94]. This is common in creation a metallic shell coating the metallic exited core of nanostructures with a large range of various morphologies such as nanospheres, nanoboxes, nanorings, nanotubes and nanocages [94,95]. Ni–Pt, Au–Ag and Cu–Ag nanoparticles are examples that are produced by galvanic displacement method [96,97,98].

#### 3.2.2. Electrochemical Synthesis‘

Electrochemical synthesis uses electricity as the main source of composite reactions. This method is mostly used in industrial applications. An electrical field is created by two electrodes. Reduction occurs at the metallic anode or with the anode itself dissolved in solution along with the metal precursor, and new metallic nanoparticles are formed at the cathode. A stabilized chemical for stabilization of fresh metal particles has to be included there. This method has advantages of controlling the nanoparticle size, imparting high purity, and being environmentally-friendly and cost-effective [1,16,30,99]. Therefore, this technique is applied in the large scale manufacture of many bimetallic (Rh–Pd, Au–Pt, Pt–Au, Ag–Au, Cu–Ag, At–Ni) and trimetallic (Pd–Fe–Ni, Pd–Ag–Cd) alloy particles [16,19,100,101,102,103,104,105,106,107,108,109,110,111].

#### 3.2.3. Hydrothermal Methods-Based Nanoparticle Synthesis

In this method, nanoparticles are synthesized in high-temperature aqueous solutions at a high vapor pressure. Although this method allows monitoring of the nanoparticle growth, and their physical and chemical properties, its disadvantages include high-temperature conditions and high-cost equipment [30,112,113]. Many bimetallic nanoparticles such as NiFe_2_O_4_ (nickel ferrite), co-doped Zn_1−x_Co_x_Mn_2_O, Ni–Cu, Au–Cu, Ag–Co, Ni–Fe, and Co–Ni nanoparticles are created using the hydrothermal method [114,115,116,117,118,119].

#### 3.2.4. Chemical Precipitation-Based Nanoparticle Synthesis

Chemical precipitation involves the formation of solids from a solution by creating a supersaturated condition or by converting the soluble material into an insoluble form via pH change, electrooxidising potential, or adding of a precipitation reagent [30,120,121]. A typical chemical precipitation method contains four stages: precipitation, flocculation, sedimentation, and solid-liquid separation [121]. This method is a single-step process that is useful in large-scale production of nanoparticles. Chemical precipitation is popularly utilized in water purification [30,120,121]. Some studies have reported the fabrication of Mg–Zn, Pd–Fe, and Fe–Ni–Ce alloy nanoparticles by the chemical precipitation method [122,123,124].

#### 3.2.5. Other Chemical Methods of Nanoparticle Synthesis

The above-mentioned methods involve synthesis in homogeneous liquids such as water or organic solvents; however, there are other synthesis methods that use the gas phase or heterogeneous phases such as sol-gel and micro-emulsion.

There are some methods by which metal nanoparticles are synthesized in a gaseous environment such as the selective catalytic reduction method and flame spray pyrolysis. In the flame spray pyrolysis method, a metal precursor solution that is sprayed is reduced by temperature, turning into a metal particle [125,126]. In the selective catalytic reduction technique, the reaction changes nitrogen oxides using a gaseous catalyst such as urea and ammonia [33,127].

The sol–gel approach involves the basic steps of hydrolysis, condensation, and drying. There are two types, aqueous sol–gel in which the solvent is water, and nonaqueous sol–gel in which the solvent is an organic solvent. This method is simple, economical, and can be processed at low temperature [67,127,128,129,130,131,132,133,134].

The micro-emulsion method, in its simple definition, is a system comprising three components: a minor droplet (dispersed phase), an immiscible solvent (continuous phase), and a surfactant that covers the droplet. Depending on the properties of the dispersed phase, continuous phase, and the hydrophilic–lipophilic balance value of the surfactant, there are many types of micro-emulsions, such as water–oil, oil–water, and water-Triton X-100 among others. The metal nanoparticles are synthesized inside droplets that can be designed to the desired size and composition. This method has been applied broadly in the synthesis of bimetallic and trimetallic alloy nanoparticles [135,136,137,138,139,140,141,142,143].

### 3.3. Biological Methods of Nanoparticle Synthesis

Since the development of nanotechnology, many approaches for nanoparticle synthesis have been discovered and improved. Most of these are chemical methods based on hazardous chemicals, enormous energy, and high temperature and form nanoparticles with limited properties. To overcome these disadvantages, green synthesis approaches, such as those based on microwave, electrochemical, hydrothermal, and sonochemical methods have been developed. Another green synthesis method that has progressed recently is based on biological sources such as plants, microorganisms, and industrial and agricultural wastes [144,145,146,147,148]. Biological synthesis has been applied to a large extent in nanoparticle production and has also been used for fabricating bimetallic or trimetallic alloy nanoparticles.

#### 3.3.1. Microorganisms to Produce Nanoparticles

**Figure 3:**
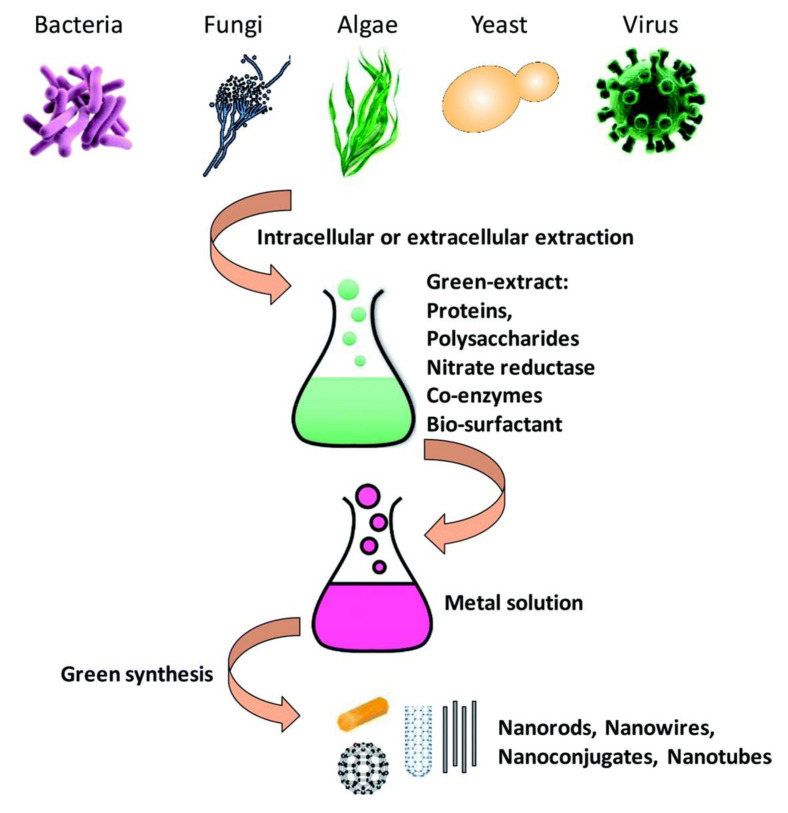
Schematic of metal nanoparticle synthesis by microorganisms [149].

Micro-sized organisms, including bacteria, fungi, yeasts, and even viruses have been considered as nano-factories to produce nanoparticles (Figure 3) because of their ability to accumulate and detoxify heavy metals via various reductase enzymes [149]. The metal reduction can be carried out in the extracellular or intracellular environment. The genes, proteins, enzymes, and biomolecules of the microorganisms play roles as reducing factors. Bacteria such as *Escherichia coli*, *Salmonella typhimurium, Listeria monocytogenes*, *Bacillus subtilis,* and *Rhodopseudomonas capsulata* [150,151,152,153,154,155,156,157] have been used to create Au–Pd, Pd–Pt, Pd–Ag, Au–Ag, Pd–Fe, Au–Fe, Pd–Au–Fe, and Cu–Ag alloy nanoparticles.

Fungi and yeasts have been used for nanoparticle synthesis. Compared to bacteria, they have some advantages such as high accumulation, high yield, easy to culture, and presence of complex proteins that help in nanoparticle synthesis. When fungi are exposed to a metal ion environment, they produce compounds or biomolecules such as naphthoquinones, anthraquinones, or nitrate reductase as reducing factors to create metal particles [144,145,158,159]. Fungi such as *Fusarium semitectum, Neurospora crassa, Fusarium oxysporum, Pleurotus ostreatus, Coriolus versicolor* and yeasts such as *Saccharomyces cerevisiae, Schizosaccharomyces, Schizosaccharomyces pombe*, and *Candida glabrata* have been used to manufacture Au-Ag and Cd-S alloy nanoparticles [160,161,162,163,164,165,166,167,168,169,170].

Viruses, which are not considered as a complete living organism, are also utilized in nanomaterial synthesis. Particularly, plant virus capsids work as a useful bio-template in nanoparticle production [171,172,173]. Some plant viruses (Cowpea mosaic virus, tobacco mosaic virus, Red clover necrotic mosaic virus) have been used in producing Fe–Pt, Co–Pt, Co–Fe, Cd–Se alloy nanoparticles [174,175,176].

#### 3.3.2. Plants as Source of Nanoparticles

Recently, plants have been explored as an option for the green synthesis of nanomaterials. It involves the application of various plant organs such as the root, stem, leaf, seed, fruit peel, and flowers and their extracts to manufacture nanoparticles. This method is eco-friendly and stable, and the created nanoparticles have potential use in biomedical and environmental applications. It is proposed that plant constituents, including protein, amino acids, organic acid, and polysaccharides, and secondary metabolites such as polyphenols, flavonoids, alkaloids, heterocyclic, and terpenoid compounds play roles as reducing agents and stabilizing factors [144,145,177,178]. Monometallic nanoparticles as well as metallic alloy nanoparticles have been manufactured using plant-based approaches. For instance, Ag–Ni, Ag–Co, Pt–Cu, Au–Ag, Ag–Cu, and Zn–Ag nanoparticles have been produced using the leaf extracts of *Canna indica*, *Alchornealaxiflora*, *Azadirachta indica, Cacumen Platycladi*, palm, *Mirabilis jalapa,* and *Moringa oleifera* [179,180,181,182,183,184,185], Au–Ag–Sr, and Fe–Ag–Pt nanoparticles were produced from the roots of coriander, *Platycodon grandiflorum* [13,186], and Au-Ag nanoparticles have been produced from the Chinese wolfberry fruit extracts [187].

Algae are small eukaryotic organisms also used for the synthesis of alloy nanoparticles; for instance, *Phaeodactylum tricornutum*, *Chlamydomonas reinhardtii*, and *Spirulina platensis* were utilized to prepare CdS and Au-Ag nanoparticles [188,189,190].

#### 3.3.3. Agricultural and Industrial Waste as Source of Nanoparticles

**Figure 4:**
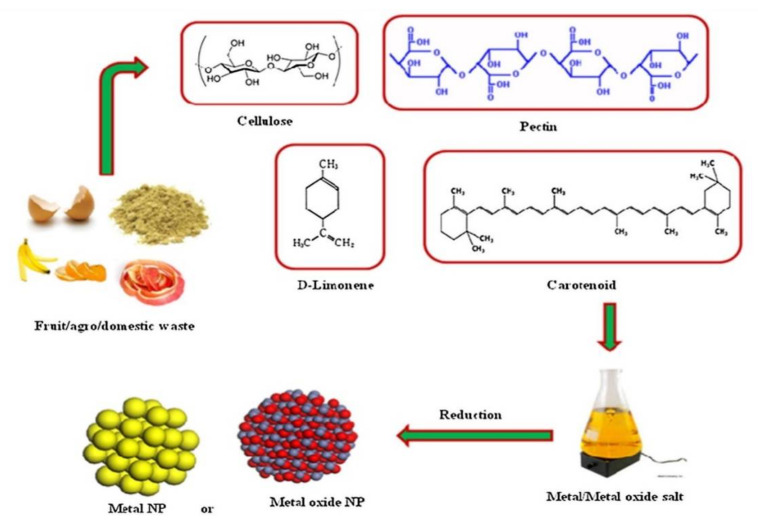
Synthesis of nanoparticles using waste materials [195].

In recent years, nanoparticles have been synthesized largely from agricultural and industrial wastes (Figure 4). Post-harvest wastes such as fruit peels, rice husk, and egg shells form approximately 80% of the biomass on the field, and industrial waste such as timber dust, sugar cane bagasse, and wild weeds including unwanted plants, herbs, or shrubs that are usually burned, can be used as biological sources for the green synthesis of nanoparticles. The use of these waste materials compared with the physical and chemical methods has benefits including reduction of using harmful chemicals, low-cost, low energy, and renewing waste material [191,192,193,194,195]. Many monometallic nanoparticles were manufactured using citrus fruit peel extract, grape waste, mango peel, rice husk, sugar cane bagasse and leaves, bamboo leaves, egg shell, and coconut shells. Moreover, Au–Ag and CdS alloy nanoparticles were also manufactured using banana peels and the otherwise useless weed *Antigonon leptopus* [196,197,198].

Typically, biological synthesis depends on pH, temperature, pressure, time, and protocol. It has plenty of advantages such as being an ecofriendly, low-cost, safe, and simple method that requires a short time. The biosynthesized nanoparticles have biocompatible characteristics and can be introduced into biological and pharmacological applications without an additional step of attaching to bioactive compounds. However, these synthesis methods also have some disadvantages due to the complicated parameters or complex constituents in plant organs; the size and shape of nanoparticles can be seldom controlled well. In some cases, the generated nanoparticles cause toxicity to the plant, and bacteria [149,199].

Nowadays, alloy nanoparticles with supports like carbon, silica substrates, and graphene sheets among others are being increasingly manufactured, thereby generating a variety of useful of nanoparticles.

## 4. Properties of Alloy Nanoparticles

The most distinctive feature of bimetallic or trimetallic alloy nanoparticles is the combination of physicochemical properties of the chemicals from which they are created. Typically, there are three main metal groups based on their characteristics: Cu, Ag, Au for plasmonic; Pd, Pt, Ru, and Rh for catalysis; and Fe, Ni, Co for magnetism. Combining two or more metals almost always increases the inherent characteristics. For example, the Au–Pt bimetallic nanoparticles composed of plasmonic Au and catalytic Pt have a hybrid property of catalytic ability that can be boosted by light [31].

### 4.1. Catalytic Properties

Many studies have shown that alloy nanoparticles are more active than monometallic nanoparticles made with the same metal. The catalytic properties of bimetallic nanoparticles depend on the structures that are different between the core–shell structure and the disordered structure and composition of alloy nanoparticles. Pt–Ni alloy nanoparticles catalyze the oxygen reduction reaction ten times faster than Pt nanoparticle [200]. Another report showed that Pt–Ru alloy and Pt–Ru core–shell nanoparticles show differences in catalytic reactions [201]. Further, among core–shell bimetallic nanoparticles, the catalytic activities are different as indicated in the study by Tsang’s group; they showed that the catalytic action of M–Pd core–shell nanoparticles (M = Rh, Pt, Ru, Au, Ag) in formic acid decomposition increased linearly with the increasing of difference in charge density between the core mental and the shell [202]. The thickness of the shell also affects the catalytic activity [203].

### 4.2. Photocatalytic Properties

Photocatalytic characteristics are a great advantage of alloy nanomaterials composed of a plasmonic metal and catalytic metal, such as Au–Pd, Ag–Pt, and Cu–Pd nanoparticles. Photocatalysis occurs with visible or ultraviolet light, which is absorbed and subsequently released as energy that facilitates catalysis. Some reports indicate that plasmons support chemical transformation. Further, thermal assistance is more effective for photocatalysis [31,204,205,206].

### 4.3. Optical Properties

Localized surface plasmon resonance (LSPR) is an optical property of nanoparticles. Among metals, Au, Ag, Cu, Pd, and Pt have attracted much attention due to their optical properties that are largely applicable in photocatalysis, biomedicine, Surface-enhanced Raman spectroscopy (SERS), and photothermal therapy. Optical properties depend on the size, shape, and composition of nanoparticles [207]. Some studies have shown that LSPR peak position, intensity, and line width is influenced by the size of nanoparticles. For example, by decreasing the size of Au nanoparticles, the emission light position changes from the NIR region to the UV region. Due to a very small size, nanoparticles can lose their LSPR and become photoluminescent [208,209,210,211,212]. In addition, LSPR sensitivity is highly dependent on the shape of nanoparticles. One study reported that the sensitivity of Au nanoparticle increased in the order of spheres, cubes, shells, rods, rattles, stars, branches and rings [31].

The optical property of bimetallic nanoparticles is strongly affected by their component metals. LSPR increases in the case of combining two plasmonic metals being resonant in the visible region (Au–Ag, Au–Cu nanoparticles), whereas it decreases or quenches if one metal in the combination is resonant in the UV region (Pd, Pt) like Ag–Pd nanoparticles. In core–shell alloy nanoparticles, LSPR is also affected by the shell metal. With increasing thickness of the shell, the LSPR peak position moves directly from the peak position of the metal forming the core to the peak position of the shell metal [31,213].

### 4.4. Magnetic Properties

Compared to monometallic nanoparticles, bimetallic nanoparticles have an extra useful feature of the magnetic property. According to Bansmann’ s group, a mixture of 3d metals (Fe, Ni, etc.) with big local magnetic moments and 4d or 5d metals (Pd, Pt, etc.) with strong spin-orbit coupling creates bimetallic nanoparticles with high magnetic moments and large anisotropy [214]. Further, Pt-based nanoparticles have additional properties such as oxidation resistance and catalysis. In summary, combination of these metals to produce alloy nanoparticles results in more effective magnetism, better stability, and additional catalytic characteristics [31,214].

## 5. Application of Alloy Nanoparticles in the Biological Field

Similar to monometallic nanomaterials, bi and trimetallic alloy nanomaterials are used in a large range of biological applications. Moreover, alloy nanomaterials show interesting synergism in the properties of the metals from which they are created. This allows alloy materials to be used more effectively. Here, we discuss the bio-application of alloy nanoparticles in imaging, diagnosis, and therapies.

### 5.1. Imaging

**Figure 5:**
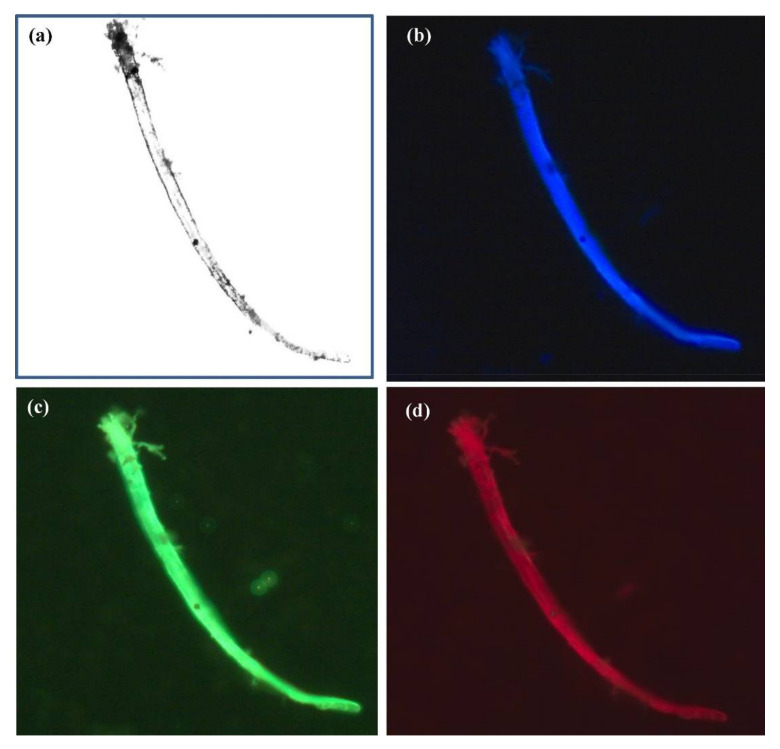
Fluorescence microscopic images of fungal cells (Alternaria sp.) treated with chicken egg white-L-Cysteine-encapsulated Zn-undoped Ag nanoclusters. Image (**a**) without chicken egg white-L-Cysteine-encapsulated Zn-doped Ag nanoclusters and images with chicken egg white-L-Cysteine-encapsulated Zn-undoped Ag nanoclusters upon the laser excitation at (**b**) 405  nm (blue), (**c**) 488  nm (green), and (**d**) 561 nm (red), respectively. The concentration of chicken egg white-L-Cys-encapsulated Zn-undoped Ag nanoclusters was 0.5 μg/mL [221].

Researchers always aim towards a better understanding of the structure as well as the function of living organisms. This requires high-quality bioimaging at various levels ranging from molecules, cell, organs, to the whole body. Microscopy and many different methods have long been used to capture the images of the cells or whole body of organisms. Nanomaterials that have optical properties are widely applied in bioimaging. Until now, there have been many studies about using plasmonic nanoparticles like Au nanoparticles and quantum dots in imaging cell components, surface species, endocytic pathways, cell cycle and apoptosis processes, cell secretion, animal organs, and microorganisms [215,216,217]. Nanoparticles acting as bioimaging probes possess characteristics such as the ability to penetrate into cells, good analytical signals, solubility and stability in relevant solvents or intracellular environments, ability to attach with functional groups for site-specific labeling, and low cytotoxicity [218]. Au and Ag-based bimetallic nanoparticles have been developed for cell imaging. For instance, Ag–Au nanoparticles or porous nanospheres combined with biomolecules exhibit enhanced optical properties, good dispersion in aqueous solution, high physiological stability, and favorable biocompatibility and were used as a label probe [219,220]; Zn doped Ag nanoclusters with L- cysteine and chicken egg white showed an increased quantum yield compared with pure Ag nanoclusters; moreover, they showed excellent stability in their role as a probe in the imaging of fungal cells (*Alternaria* sp.) [221] (Figure 5). Cu-doped Au nanoclusters exhibited fluorescence intensity that decreased linearly with increasing Cu concentration but exhibited higher photostability than Rhodamine 6G (conventional fluorescent dyes) at 24 h in ex vivo as observed in self-illuminating NIR images of major organs (tumor, heart, liver, spleen, lungs, and kidneys) from U87MG tumor-bearing mice [222].

### 5.2. Diagnosis

#### 5.2.1. Biomedical Imaging

Bioimaging is used for the recognition of shapes, structures, and pathways in organisms as well as in disease diagnosis, especially in cancer and tumor detection. Gold and iron-based nanoparticles and quantum dots are used in many biomedical imaging techniques such as magnetic resonance imaging (MRI), computed tomography (CT), photoacoustic (PA) imaging, high-order multiphoton luminescence (HOMPL) microscopy, contrast-enhanced dual-energy mammography (DEM), and so on for cancer and tumor detection [223,224].

To overcome the disadvantages of nanoparticles like brightness, alloy nanoparticles have been demonstrated to possess superior qualities in biomedical imaging. Iron-based alloy nanoparticles such as Fe–Ni, Fe–Pt are utilized in magnetic resonance imaging as potential contrast agents that show a high magnetic or superparamagnetic property, along with low toxicity in living cells [224,225,226,227].

According to the study by Cormode and group, Au–Ag alloy nanoparticles are applied in dual-energy mammography (DEM) or computed tomography (CT) as imaging probes for breast cancer screening [228]. The gold in the alloy nanoparticle reduces the leaching of silver from the particle and increases biocompatibility. In an in vivo experiment, the Au–Ag nanoparticle showed a clear contrast in the tumor when analyzed by the DEM and CT techniques. Therefore, Au–Ag nanoparticles are a prospective contrast agent for breast cancer detection by DEM and CT [228].

#### 5.2.2. Sensors

The superb plasmonic property of Ag- or Au-based alloy nanoparticles allows their applications as sensors using the SERS technique or fluorescence with a variety of detected targets like metal ions, chemicals, and biomolecular targets [229]. For example, using a keratin template, Ag-Au nanoclusters have been fabricated for mercury ion detection. The alloy material shows approximately five-fold higher fluorescence than that with Au nanoclusters with keratin. This proved that Ag addition supports the fluorescence intensity and makes the alloy cluster more stable. Thereby, mercury ions were detected in a wide range with low detection limits [230]; AuM (M can be Pd, Pt, Rh) nanoparticles show remarkably enhanced hydrogen peroxide (H_2_O_2_) detection when compared with Au, Pt, Rh, Pd monometallic nanoparticles [231], and Au–Ag, Ag–Pd, Ag–Pt bimetallic nanoparticles are utilized in detection of biomolecules such as glutathione, cysteine, endonuclease, L-cysteine, and adenine with ultra-bright fluorescence or SERS intensity [232,233,234,235,236].

Immunofluorescence technology using quantum dots as beneficial reporters exhibit properties such as sensitivity, high specificity, and fast results due to the characteristics of quantum dots including higher photoluminescence and quantum yields, higher optical and chemical stability, and broader emitting range. Especially, alloy quantum dots have improved photoluminescence intensity and stability compared to standard quantum dots. Therefore, they have been widely used in clinical diagnosis, clinical analysis, and cancer detection [237,238,239]. For instance, ZnSe/CdS/ZnS core–shell quantum dots have been used in the detection of C-reactive protein, an early indicator of infection and autoimmune disorders [237]; bovine serum albumin-doped CdS quantum dots have been used in detecting human IgG1, a low-abundance protein [240]; Cu:ZnInS/ZnS quantum dots have been used in tetanus antibody detection [241]; CdTe quantum dots are used for detecting α-fetoprotein, a tumor marker [242]; and S- and N–Co-doped carbon quantum dots have been used in detecting clenbuterol, a feed additive for livestock and poultry [239].

#### 5.2.3. Catalyst

Nanoparticle-based catalysts are structured with catalytically efficient metals such as Pd, Ni, and Pt. Similar to other alloy nanomaterials, catalysts like Pd–Ru and Pd–Co, made of bimetallic nanomaterials have excellent hybrid catalytic characteristics and exhibit photocatalysis when combined with metals with optical properties like Pt–Au [243,244]. Bimetallic catalysts thus show higher catalytic activity and longer stability than monometallic catalysts.

**Figure 6:**
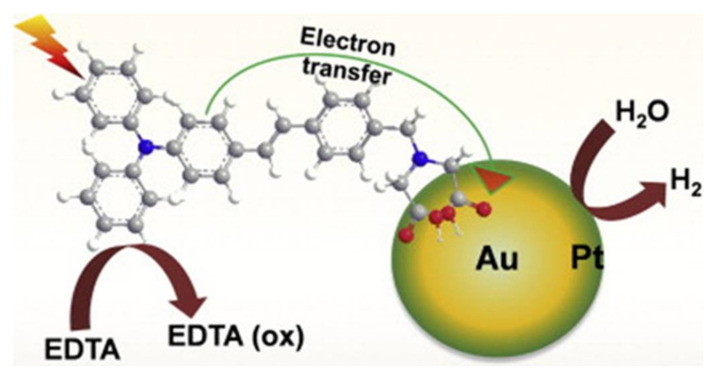
Schematic representation of electron transfer from a triphenylamine derivative, 2,2′-(4-(4-(diphenylamino) styryl) benzylazanediyl) diacetic acid (TPAD) sensitizer into an Au core–Pt shell nanoparticle and photoinduced hydrogen evolution under light irradiation [248].

For example, between Pd-Ru nanoparticles and Ru nanoparticles produced by *Bacillus benzeovorans,* Pd-Ru nanoparticles show better catalytic activity than Ru nanoparticles in converting 5-hydroxymethyl furfural (5-HMF) to the fuel precursor 2,5-dimethyl furan (2,5-DMF) [243]. In methanol and ethanol electrooxidation, Pd-Co nanoparticles supported on graphene show a slight decrease in catalysis but show strong stability, long term use, and magnetic property that allows easy separation from a mixture compared with Pd nanoparticles supported on graphene. Further, Pd–Fe, Pd–Zn, Ni–Fe, Pt–Fe, and Ag-Pd nanoparticles exhibited more effective catalysis of chlorinated organic solvents and chlorinated aromatic compounds [245,246]. The photocatalytic activity of CuAu-ZnO-Graphene nanocomposite and Au–Pt-TPAD (triphenylamine derivative, 2,2′-(4-(4-(diphenylamino) styryl) benzylazanediyl) diacetic acid (TPAD)) (Figure 6) was higher than the catalytic activity of the respective monometallic nanomaterial [247,248].

Trimetallic nanomaterials show superior catalytic activity compared to bimetallic or monometallic nanoparticles. Various studies have reported that Ag–Au–Pt and Au-Pd-Pt trimetallic nanoparticles possessed superb catalytic activity in methanol oxidation that are not seen with Pd–Pt, Au–Pt, and Pt–C bimetallic nanoparticles or with Pt and Au monometallic nanoparticles [17,249,250].

### 5.3. Therapy

#### 5.3.1. Drug Delivery and Cancer Therapy

In therapy, the aims of drug delivery include precise targeting (target infected cells but not healthy cells), therapeutic efficacy, and low cytotoxicity, whereas conventional drug delivery systems pose challenges such as poor specificity, low therapeutic activity, and toxicity. Modern drug carriers exhibit satisfactory characteristics of specific targeting, controlled drug release, and less toxicity, and are called smart carriers [251,252,253]. Drug carriers are fabricated using organic materials (polymeric micelles, solid lipid, liposome, and dendrimer) or inorganic materials (silica nanoparticles, Au nanoparticles, magnetic nanoparticles, quantum dots, carbon nanotubes, and nanographene) [253,254] and are discussed in detail in this review.

**Figure 7:**
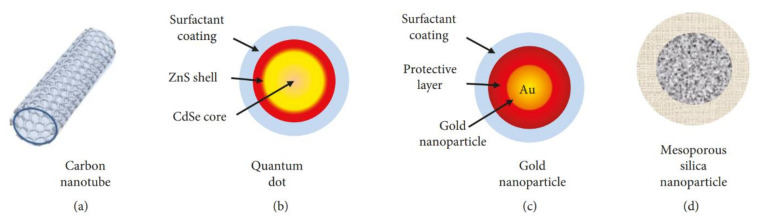
Example of the most employed inorganic nanocarriers: (**a**) carbon nanotubes, (**b**) quantum dots, (**c**) gold nanoparticles, and (**d**) mesoporous silica nanoparticles [253].

Inorganic carriers are typical core–shell structures (Figure 7). Their core part can be made of gold, quantum dots, or silica, whereas the shell part contains organic polymers, ligands that provide biocompatibility, and protection that help carriers to easily infiltrate the biological environment of the human body. The properties of carriers are tuned by composition, shape, size, and surface modifications. In addition, they have the novel property of controllable delivery and release in response to a variety of stimuli such as light, pH, temperature, magnetic force, enzyme reaction, among others. They can carry many kinds of therapeutic molecules including anticancer drugs (doxorubicin, paclitaxel) RNA, DNA, proteins, and antibodies [253,255].

Besides being a drug carrier, these inorganic carriers can also act as therapeutic agents, especially in cancer therapies such as radiotherapy, magnetic hyperthermia therapy, photodynamic therapy, and photothermal therapy [254].

Gold-based nanoparticles are a good candidate for cancer therapy due to their brilliant properties. Gold nanoparticles are nontoxic for some human cells, which is beneficial for intracellular treatment. The negative charge helps gold nanoparticles acquire bio-functionality in conjugation with various functional molecules such as DNA, protein, and antibodies. Further, gold is a radiosensitizer that can convert light to heat that can be used in cancer treatment [253,254]. One report said that the irradiation of gold nanoparticles induces transient changes locally of cell membrane permeability in biological environment that can support the design of new drug delivery systems [256]. Another report also showed that Ag–Au alloy nanoparticles exhibited hepatoprotective activity against diethylnitrosamine (DEN)-induced liver cancer in a Sprague Dawley rat model [257]. Gazouli et al. demonstrated that Ag–Au (ratio 3:1) alloy nanoparticles synthesized by a chemical reduction method could prevent excessive tryptophan-induced apoptosis in cancer cell lines, HCT116 (colon cancer cells), 4T1 (highly metastatic breast cancer cells), and HUH7 (hepatocyte-derived carcinoma cell line) via p53, CASPASE-3, and BAX/BCL-2 pathways [258]. Another study about the inhibitory effect of the ordered topology of Ag–Au alloy nanoparticles on mouse Lewis lung carcinoma indicated that Ag core–Au shell type particles with a 1:1 ratio possessed the best antitumor activity and lowest toxicity among other types of alloy nanoparticles including, Ag core–Au shell and Au core–Ag shell particles with different ratios [259].

Quantum dots are semiconductors with fluorescence properties that allow them to be used for real time drug delivery and as photosensitizers. With surface modification using tumor recognized molecules and drugs, quantum dots can move to target tumors and act as a supporting energetic agent in photodynamic therapy, a method that combines light and photosensitizers to generate oxygen species and promote tumor suppression [254,260,261,262]. Quantum dots are frequently used in a complex like with graphene and photosensitizer such as chlorin e6 [261,263]. Martinenko et al. reported that the complex ZnSe/ZnS quantum dots with chlorin e6 showed two-fold enhancement of photodynamic destruction in Ehrlich ascites carcinoma cells compared to that with free chlorin e6 molecules, and approximately 50% fluorescence resonance energy transfer from QDs to the chlorin e6 molecules [264].

Hyperthermia therapy is based on heat. The temperature in tumor cells is increased to 40–45 °C for inducing certain pathways including apoptosis. Magnetic nanoparticles can be heated under the magnetic field leading to direct killing of cancer cells, or by releasing the carried drug to kill the cancer cells [265,266]. Many kinds of magnetic nanoparticles (superparamagnetic iron-oxide, Fe_3_O_4_ or Fe_2_O_3_, Mn–Zn, and Fe–Au nanoparticles) are created and surface-modified using aminosilane, antibodies, PEG-phospholipids, or cyclic tripeptide of arginine-glycine-aspartic acid for the treatment of glioblastoma multiforme tumors [267,268,269,270,271]; or in the activation of reactive oxygen species via the p53 pathway in killing HepG2 human hepatocellular carcinoma and A549 human lung adenocarcinoma cells [272].

#### 5.3.2. Antibacterial Activity

Since their discovery, antibiotics have been used in the treatment of diseases caused by bacteria. Unfortunately, due to the broad use of various antibiotics, drug-resistant bacteria develop and quickly become a challenge for human health. Therefore, novel methods for bacterial treatment need to be identified. Recently many studies have shown that metallic nanomaterials possess antibacterial activity. For instance, gold, silver, copper titanium, and iron nanoparticles exhibit physicochemical properties and antibacterial effects that depend on their size, structure, shape, and surface modification. Au nanoparticles show antibacterial properties at 2 nm size and Ag nanoparticles offer higher antibacterial properties with triangle-shaped nanoplates compared to other shapes. The underlying mechanisms of bactericidal effects of nanoparticles are diverse; metal nanoparticles are proposed to hinder bacterial growth by affecting the bacterial cell surface, entering into bacterial cells and inducing reactive oxygen species (ROS), damaging DNA, proteins, or inhibiting enzyme activities [273,274,275].

Among the metals mentioned above, silver plays a dominant role due to its antibacterial effects. Further, alloy nanoparticles combining silver with other metals exhibit better antibacterial properties; for instance, Cu–Ag alloy nanoparticles show more effective antibacterial properties than pure Ag or Cu nanoparticles in a resistant *Escherichia coli* strain (DH5a) and *Staphylococcus aureus* strain (BB255) [276,277]. In an experiment showing the benefit of combining Ag, Pt and Au, with the support of an Au core, the strong NIR SPR response could be transferred to the Ag–Pt alloy shell, and the alloy material endowed extra light-enhanced effects in its properties including overcoming bacterial resistance [278]. Further, Ag was added to a Co–Cr alloy material in an implant to improve the antibacterial characteristics of the material [279]. In addition to Ag-based alloy nanomaterials, other metal alloy nanomaterials such as Cu–Pt overcame bacterial resistance based on their peroxidase-like activity that can catalyze H_2_O_2_ and generate hydroxyl radicals, and inhibit bacterial growth [280].

#### 5.3.3. Potential Vaccine Adjuvant

In vaccination, adjuvants accelerate antigen-specific immune responses. Adjuvants are necessary for the development of robust immune responses. Novel adjuvants are expected to have characteristics like induction of immunization against weak antigens, production of broadening immune response against pathogens with antigenic drift, and nontoxicity [281]. Recently, nanoparticles have also shown their potential as novel vaccine adjuvants. While being a vaccine carrier, nanoparticles enhance immune responses induced by vaccines as well. The immune activation effect of metallic nanoparticles is influenced by their size, shape, structure, crystallinity, surface modification, and ligands [281,282]. For example, aluminum hydroxide nanoparticles (112 nm) show stronger vaccine adjuvant activity than microparticles (9 µm) [283]. In an in vivo experiment performed in mice, gold nanoparticles coated with West Nile virus envelop protein induced a stronger antibody response with the 40 nm sphere shape than with the 20 nm sphere, cube, or rod-shaped nanoparticles [284].

Until now, only monometallic nanoparticles based on aluminum, gold mesoporous silica, iron, and nickel have been utilized as vaccine adjuvants. The use of bimetallic alloy nanoparticles in medical applications remains to be explored in future studies.

## 6. Conclusions and Future Perspectives

The above discussions provide compelling evidence that bimetallic or trimetallic alloy nanoparticles are more advantageous than monometallic nanoparticles in many fields because of their enhanced properties. The synergistic effect which arises from the combination of two or three metals contributes to these properties. This special characteristic creates multifunctional alloy nanoparticles that can be applied in various fields. Alloy nanoparticles also show other potential abilities. Using nanoparticles with antibacterial activity to inhibit antibiotic-resistant bacteria is a topic for prospective studies. The role of nanoparticles as vaccine adjuvants is still in the early stages of research. Thus, an improvement in their existing properties promises superior advantages of using alloy nanoparticles in the future.
